# Proline Metabolism in Cell Regulation and Cancer Biology: Recent Advances and Hypotheses

**DOI:** 10.1089/ars.2017.7350

**Published:** 2018-12-27

**Authors:** James M. Phang

**Affiliations:** Mouse Cancer Genetics Program, Center for Cancer Research, National Cancer Institute at Frederick, NIH, Frederick, Maryland.

**Keywords:** proline cycle, senescence, metastasis, resistance to oxidative stress, redox signaling, pyridine nucleotides

## Abstract

***Significance:*** It is increasingly clear that proline metabolism plays an important role in metabolic reprogramming, not only in cancer but also in related fields such as aging, senescence, and development. Although first focused on proline catabolism, recent studies from a number of laboratories have emphasized the regulatory effects of proline synthesis and proline cycling.

***Recent Advances:*** Although proline dehydrogenase/proline oxidase (PRODH/POX) has been known as a tumor protein 53 (P53)-activated source of redox signaling for initiating apoptosis and autophagy, senescence has been added to the responses. On the biosynthetic side, two well-recognized oncogenes, *c-MYC* and phosphoinositide 3-kinase (*PI3K*), markedly upregulate enzymes of proline synthesis; mechanisms affected include augmented redox cycling and maintenance of pyridine nucleotides. The reprogramming has been shown to shift in clonogenesis and/or metastasis.

***Critical Issues:*** Although PRODH/POX generates reactive oxygen species (ROS) for signaling, the cellular endpoint is variable and dependent on metabolic context; the switches for these responses remain unknown. On the synthetic side, the enzymes require more complete characterization in various cancers, and demonstration of coupling of proline metabolites to other pathways may require studies of protein–protein interactions, membrane transporters, and shuttles.

***Future Directions:*** The proline metabolic axis can serve as a scaffold on which a variety of regulatory mechanisms are integrated. Once understood as a central mechanism in cancer metabolism, proline metabolism may be a good target for adjunctive cancer therapy.

## Introduction

Studies of cancer metabolism and its reprogramming have focused on energy production and the allocation of carbons for cell mass. The Warburg effect, that is, the shift from oxidative phosphorylation to glycolysis, was a major theme. Recent efforts have explored the metabolism of amino acids, especially the nonessential amino acids (NEAA), emphasizing their regulatory roles (80, 83, 99). It is likely that these mechanisms may have their origin in prokaryotes; layers of networks, signaling factors, transcriptional factors, and so on were then superimposed and selected by evolution. In the usual tissue culture studies, the comparison of steady-state differences between normal and cancer cells may not readily reveal these multilayered metabolic mechanisms. They may be evident only under stress conditions and may be affected by the microenvironment. For certain mechanisms, the metabolic programming of embryonic stem cells (ESCs) may be a good model.

Although recent reviews have discussed the role of metabolism in cancer (3, 72), several points deserve emphasis. First, much of the data is based on studies in cultured cells under various conditions. Since culture medium was originally formulated to maximize cell yield (20, 71), various nutrients were at concentrations bypassing regulatory mechanisms (15, 31). Another consideration is that reprogramming may be a process dependent on temporospatial factors (14). Steady-state differences may not exist or they may not be susceptible to pharmacologic targeting. It may be the early transient metabolic events in reprogramming with clonogenesis (86) or metastases (22), which are vulnerable to intervention.

A special role for proline in metabolic regulation is now accepted (3, 67, 72), and recent work not only showed that proline metabolism is critical in cancer reprogramming (22, 48, 86) but also established its clinical relevance (17). Unlike other amino acids, proline has its α-amino group within a pyrrolidine ring, and thus it is the sole proteinogenic secondary (imino) amino acid and has its own metabolic pathways (1). Proposed over three decades ago (74), the proline regulatory role has found support and elaboration from numerous laboratories over the last decade (46). Historically, the earlier studies were based on experimental models using reconstituted organelles and/or purified proteins that showed the function of a proline cycle, which mediated redox transfers (29, 74). An important direction was provided by the discovery that proline dehydrogenase/proline oxidase (PRODH/POX) was encoded by tumor protein 53 (P53)-induced gene 6 (PIG-6) (82), supporting the hypothesis that the proline metabolic system was mobilized during stress, and this was extended to include a variety of stresses (78).

A series of observations established that PRODH through an ROS-mediated mechanism was an important signaling pathway for apoptosis (53, 54) as well as autophagy (47, 105), depending on the upstream signaling mechanism as well as coexisting metabolic conditions. Although previous studies emphasized proline catabolism, recent findings show that the synthetic components of the proline cycle play an important role. Unlike the degradative pathway with PRODH induced by stress signals, that is, P53, peroxisome proliferator-activated receptor γ (PPARγ), and AMP-activated protein kinase (AMPK) (75), the synthetic pathway is robustly upregulated by oncogenes, that is, *c-MYC* (49) and phosphoinositide 3-kinase (*PI3K*) (48). Importantly, the differential functions of three isozymes of pyrroline-5-carboxylate (P5C) reductase were identified (16). Although there is much more to be elucidated about this metabolic regulatory system, its role in cancer is now established and has been recognized in recent reviews (3, 67, 72).

## New Discoveries in Proline Metabolism

During the last few years, a number of laboratories using a variety of experimental approaches have reported novel effects of the proline axis in cancer. These articles showed the following. (i) In tumor tissue, proline may be channeled into functions at the expense of protein synthesis (55, 56). (ii) PRODH/POX provides redox-dependent signaling for several processes; using differential transcriptomics, workers identified its role in initiating senescence (64, 65). (iii) Not necessarily due to disruption of protein synthesis, the inhibition of proline synthesis interrupts cell proliferation (35). (iv) Proline starvation induces ER stress and deregulates mechanistic target of rapamycin complex 1 (mTORC1)/4 erythrocyte binding protein 1 (4EB1) signaling in clonogenesis (86). (v) Proline biosynthesis may be linked to the maintenance of pyridine nucleotides by the salvage pathway (48). (vi) pyrroline-5-carboxylate reductase (PYCR)1/2 bind to RRMB2, a subunit of ribonucleotide reductase (RR), and are linked to its reactive oxygen species (ROS)-dependent regulation (39). (vii) PRODH/POX is linked functionally and physically to succinate dehydrogenase in mitochondrial complex II (30). (viii) When studied in three-dimensional (3D) spheroids, breast cancer cells show an upregulation of PRODH and cycling of proline (22). (ix) Data from tumor tissue from patients with breast cancer have been analyzed focusing on P5C reductase (PYCR1). This is the first study correlating PYCR1 with tumor invasiveness and clinical outcome (17). Now that the relevance of the proline metabolic axis in cellular regulation and cancer is firmly established, we can focus on specific mechanisms in an attempt to understand the basis for these newly discovered effects.

This review focuses on new findings, although a brief review of previous work necessarily is included to make mechanisms understandable; for more complete treatment, we refer the reader to previous reviews (77). Importantly, discoveries from older work, which previously were without physiologic correlation, are now the basis for interpreting new findings. Although this review emphasizes cancer in humans, the reader should be aware of the numerous publications on proline metabolism in plants (62, 108), prokaryotes (87), insects (28), and protozoans (42). Recent reviews have shown that the proline-dependent mechanisms may be universal in the biome (5, 46).

## Overview of Proline Metabolic Pathways

**FIG. 1.**
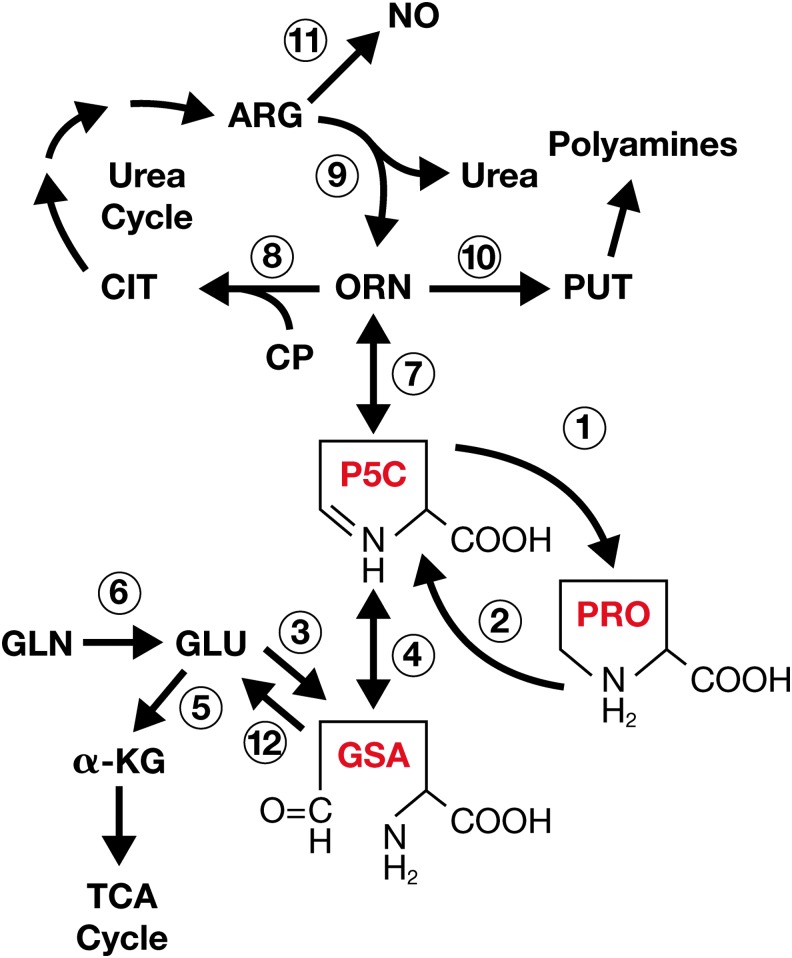
**Proline metabolic pathway.** **1**, pyrroline-5-carboxylate reductase; **2**, proline dehydrogenase/proline oxidase; **3**, pyrroline-5-carboxylate synthase; **4**, spontaneous; **5**, glutamate dehydrogenase; **6**, glutaminase; **7**, ornithine aminotransferase, **8**, ornithine transcarbamylase; **9**, arginase, **10**, ornithine decarboxylase, **11**, nitric oxide synthase; **12**, pyrroline-5-carboxylate dehydrogenase. α-KG, α-ketoglutarate; ARG, arginine; CIT, citrulline; CP, carbamoyl phosphate; GLN, glutamine; GLU, glutamate; GSA, glutamic-γ-semialdehyde; NO, nitric oxide; ORN, ornithine; P5C, Δ^1^-pyrroline-5-carboxylate; PRO, proline; PUT, putrescine; enzymes; TCA, tricarboxylic acid.

Some of the aforementioned findings will attract readers unfamiliar with the proline metabolic pathways; a brief introduction to the area will provide the foundation on which interpretations and hypotheses are based. The pioneering work of Harold Strecker (73) and Elijah Adams (1) established the metabolic scheme and described the enzymes mediating these pathways (Fig. 1); a regulatory role for proline metabolism was proposed over three decades ago (74). The central intermediate is pyrroline-5-carboxylate, which is the immediate precursor as well as first degradative product of proline. P5C can be synthesized from glutamic acid by P5C synthase (33) and from ornithine by ornithine aminotransferase (OAT) (93); the open-chain form, glutamic-γ-semialdehyde, is in tautomeric equilibrium with the cyclized form (2). The conversion of P5C to proline is mediated by P5C reductase (see the P5C reductase section below), but the oppositely directed step from proline to P5C is catalyzed by PRODH/POX, an enzyme tightly bound to mitochondrial inner membranes (38); P5C can be recycled back to glutamate by pyrroline-5-carboxylate dehydrogenase (P5CDH) (94).

It was recognized by early workers that P5C is an obligate intermediate in the metabolic interconversions between the tricarboxylic acid (TCA) cycle and urea cycle (1). This location in intermediary metabolism may allow the proline metabolic axis to mediate a number of regulatory functions (74). The bifunctionality of P5C as a precursor and product of proline led to the proposal of a proline or proline-P5C cycle (74, 81). Using isolated cellular components, workers showed that redox potential could be transferred from glucose to oxygen by the cycling of proline and P5C (29). Although these studies suggested the functioning of the proline cycle, its physiologic function remained poorly understood. To provide context for recent findings, the enzymes of the pathway are briefly discussed, but PRODH/POX and PYCR1/2/L the enzymes of the proline cycle are emphasized. More complete descriptions are available elsewhere (77).

### Proline dehydrogenase/proline oxidase

The enzyme that oxidizes proline to P5C is tightly bound to mitochondrial inner membranes (30, 37, 38) and is linked to site II of the mitochondrial electron transport chain (30, 61) with a flavine adenine dinucleotide at the active site, which transfers electrons from proline to coenzyme Q (30, 95); at site III, proline-derived electrons have two dispositions. They can be transferred to cytochrome c, which is oxidized at complex IV with electrons transferred to O_2_ to form H_2_O. On the contrary, proline-derived electrons can directly reduce dissolved oxygen at complex III to form superoxide (23, 30). Since complex III has access to both the matrix space and the intermembrane space, ROS can evolve in the mitochondrial matrix or in the intermembrane space to be transferred into the cytosol as a putative redox signal (9, 89).

**FIG. 2.**
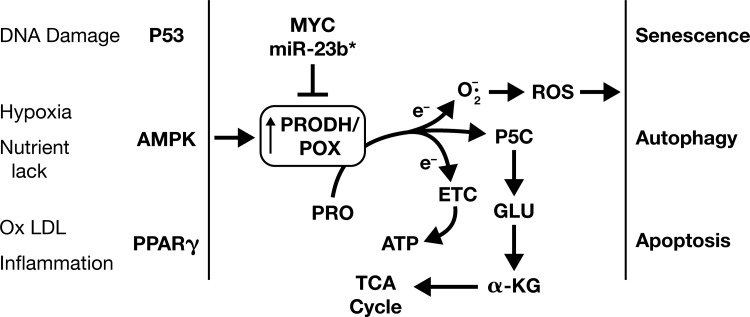
**PRODH/POX-mediated signaling.** AMPK, AMP-activated protein kinase; ETC, electron transport chain; MYC, myelocytomatosis oncogene cellular homologue; PPARγ, peroxisome proliferator-activated receptor gamma. PRODH/POX, proline dehydrogenase/proline oxidase; ROS, reactive oxygen species.

The catalytic activity of PRODH/POX can be regulated by intermediates of glucose metabolism (37), suggesting that proline can serve as a source of energy when glucose and glutamine are inadequate. Interestingly, PRODH/POX activity or protein is not found in all tissues, and differential expression is complex and mediated by a variety of mechanisms (78). The underlying theme is that *PRODH/POX* is upregulated under conditions of stress from various sources (80) (Fig. 2) and downregulated by proliferative signals (49). The recent discovery that PRODH/POX in breast tumors is upregulated during metastatic reprogramming is of considerable interest (22) and shows that the pattern of expression is linked to specific transitions.

Polyak first designated *PRODH/POX* as *PIG-6* (P53-induced gene–6) (82), which is rapidly and robustly induced in colorectal cancer cells infected with adenoP53. The organization of the *PRODH/POX* genome includes unusual features with binding sites for P53 located in both promoter and intronic regions (84). Another unusual feature is that PRODH/POX expression in the human brain may be regulated by a human-specific endogenous retrovirus (*hsERV*) as an enhancer (90). PRODH/POX can generate ROS and function as a tumor suppressor gene (18, 32, 52); the progression of tumors requires that the suppressor activity of PRODH be downregulated.

In human digestive tract and kidney tumors, the level of proline oxidase shown by immunohistochemistry is markedly decreased compared to the corresponding normal tissue in nearly 80% of those tested (52). Oncogenes such as MYC downregulate PRODH/POX expression and this may be mediated by microRNAs. One particular species, miR-23b*, specifically decreases translation by a sequence-specific binding to the 3′-untranslated region of PRODH/POX messenger RNA (51). We found that this oligonucleotide is the sibling of the common transcript from miR-23, miR-23a/b, which regulates the expression of glutaminase (27). Although miR-23b* may not be the only microRNA regulating the expression of *PRODH*, the inactivation of miR-23b* in patients remains a potential therapeutic approach.

### P5C dehydrogenase

The second step in proline degradation is the conversion of P5C back to glutamate in an NAD^+^-dependent step. Pyrroline-5-carboxylate dehydrogenase (P5CDH) is localized to mitochondria matrix and is critical in the anaplerotic role of proline released from proteins, for example, collagen. An inborn error at this step is responsible for Type 2 hyperprolinemia (94). Product glutamate can enter the TCA cycle as α-KG, thus allowing proline, either released by matrix metalloproteinases or by the import of free proline, to be used for energy and anaplerosis. We discuss “the proline cycle” in greater detail, and in the context of the cycle, P5CDH may play a role as an exit for intermediates from the proline cycle.

### P5C synthase

The two-step conversion of glutamic acid to glutamic-γ-semialdehyde is catalyzed by a single gene product, which first activates the gamma carbon with adenosine triphosphate (ATP) forming γ-glutamyl phosphate followed by NADPH-dependent reduction to produce glutamic-γ-semialdehyde, which spontaneously cyclizes to P5C (1). The human P5CS can be alternately spliced to form two mature transcripts, which differ in their sensitivity to inhibition by ornithine (33). The short form is localized to the small intestine and catalyzes the conversion of glutamate to P5C, ornithine, and subsequently to arginine. The long form is found in most other tissues and is insensitive to regulation by ornithine. A detailed description of P5C synthase can be found elsewhere (33).

### P5C reductase

The likelihood of isoforms of PYCR was suggested by earlier studies characterizing lymphocytic leukemia cells (REH), which had partial proline auxotrophy (57). Compared to normal lymphocytes (LHN13), REH exhibited deficiency of NADH-mediated activity, whereas the NADPH-mediated activity was comparable to LHN13. In addition, PYCR purified from human red cells to single-band homogeneity had high NADPH-mediated activity and the affinity for NADPH was 10-fold greater than for NADH, characteristics distinctly different from the activity found in fibroblasts and later to be shown to be PYCRL and PYCR1, respectively. With NADPH (60), the erythrocyte enzyme exhibited a Km for P5C, which was fivefold lower than with NADH (60). These findings suggested that the enzyme in human red cells functioned as a P5C-dependent NADPH dehydrogenase rather than to synthesize proline.

Human P5C reductase was cloned by a complementation strategy using yeast lacking PYCR (19). Eventually, three genomic sequences were identified coding for three proteins designated PYCR1, PYCR2, and PYCRL (PYCR3). Using cultured melanoma cells and protein products produced by *in vitro* translation, De Ingeniis *et al.* initiated studies characterizing the location and function of the three isoforms (16). They showed that PYCR1 and PYCR2 preferentially use NADH and are located in mitochondria, whereas PYCRL prefers NADPH, is located in the cytosol, and is insensitive to inhibition by product proline. Although proline could inhibit the catalytic activities of both PYCR1 and 2, the sensitivity to proline is greater by fivefold with PYCR2 suggesting that PYCR2 is regulated to produce proline (16). These workers suggested that ornithine may be the main precursor for P5C utilized by PYCRL, whereas glutamine serves as the precursor for PYCR1/2. These conclusions, however, were drawn from studies in a cell line that had high expression levels of PYCRL. Interestingly, in cells defined as exogenous proline dependent (EPD), Sahu *et al.* showed that the critical enzymes deficient were pyrroline-5-carboxylate synthetase (P5CS) and PYCRL (86) (see below). Since PYCR1/2 are the main producers of proline, the PYCRL link is of special interest. It may well be that the location and preferred use of cofactor may be dependent on cell type and metabolic context, for example, regulation by P53 or MYC.

Structural studies on human PYCR1 have shown that the holoenzyme is a decamer in the shape of “donut,” a protein configuration frequently associated with membranes (59). Whether this is a feature responsible for the multiple linkages of PYCR1 remains to be explored. A recent report disagreeing with previous observations emphasized that the NADH-binding site of PYCR1 is in a classic Rossmann fold (10).

### Ornithine aminotransferase

**FIG. 3.**
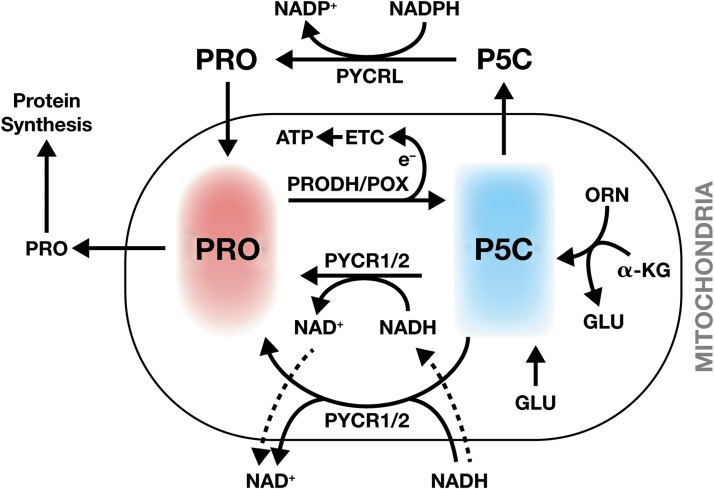
**Hypothesis for proline cycle revised.** The cycle has been revised according to locations of the enzymes. The *colored areas* are for emphasis and do not represent specific locations. The *dotted arrows* represent putative shuttle systems, for example, malate/aspartate shuttle. PYCR1/2/L, pyrroline-5-carboxylate reductase 1/2/L.

The reversible transamination of P5C by OAT to form ornithine or *vice versa* is the bridging pathway between proline and arginine and is the sole pathway transferring carbons between the tricarboxylic acid cycle and the urea cycle. An inborn error in humans is known in which deficiency of OAT results in gyrate atrophy of the choroid and retina, a disorder resulting in blindness by the fifth decade of life (93). Some have proposed that a knockdown (KD) of P5CS, the enzyme producing P5C from glutamate, halts proliferation because the cells are deficient in ornithine/arginine as well as polyamines (96). However, recent work shows that P5CS functions primarily to produce P5C as substrate for the P5C reductases (48). An interesting finding from De Ingeniis *et al.* (16) suggests that OAT catalyzes the formation of P5C for the cytosolic PYCRL. The conclusion was drawn from experiments with ^13^C-glutamine enrichment, but the cell used had markedly higher levels of PYCRL than other cells studied (4) (Fig. 3B). In addition, OAT-like P5CS is located in mitochondria, and the mechanisms channeling P5C to the cytosol as substrate for PYCRL are not known. In cells expressing MYC, the oxidized arm of the pentose phosphate pathway (oxPPP), linked to PYCRL through NADP^+^/NADPH, was markedly decreased when P5CS was knocked down (48). Thus, in these cells, the production of NADP^+^ by PYCRL was using glutamate-derived P5C for substrate. Nevertheless, differential linkages in various cell types may exist, and regulated channeling of intermediates is a likely possibility (87).

## The Proline Cycle

The proline metabolic axis has been mainly considered a nutritional interchange for metabolic intermediates to compensate for deficiencies in interconnected pathways. Whatever the source and endpoint of such metabolism, however, investigators emphasized specific end products, proline as a source of arginine and/or polyamines on the one hand (96, 98) and α-KG for anaplerosis on the other (1). In recent years, it became apparent that such interpretations based solely on carbon transfers are inadequate to explain some of the findings; we introduced the concept of “parametabolic” regulation (77, 79). In this hypothesis (see the Proline Synthesis and Cell Regulation section below), “the journey is the destination.” It is not the apparent product of the interconversions but the mediators of the metabolic journey that may play a critical role in regulation. A recent review on serine metabolism emphasized that the conversion of serine to glycine was a source of one-carbon transfers and not used to synthesize glycine *per se*. This is a good example of parametabolic regulation (41). These features of the proline cycle and the regulatory axis were reviewed, but the new findings and the interfacing with diverse disciplines have attracted scientists unfamiliar with the basics of proline metabolism. Thus, a brief reprise is appropriate.

That P5C is not only the committed precursor but also the immediate degradative product of proline has been noted by a number of investigators, and a metabolic cycle was proposed (74). However, the demonstration of a metabolically functioning cycle presented technical challenges, especially in the late 1970s and early 1980s when the cycle was proposed. However, using isolated cellular components and substrates labeled with ^14^C or ^3^H, it was possible to trace carbon products as well as redox transfers (29, 74, 81). Recently, both the cycling of proline and the parametabolic hypothesis have been supported by work from several laboratories (22, 48).

From the proline degradation half cycle mediated by PRODH/POX, the products are pyrroline-5-carboxylate, ROS, and ATP, produced by electrons passed through the electron transport chain (ETC) (Fig. 3). On the contrary, the synthetic half of the cycle involves the conversion of P5C to proline by one of three distinct isozymes of P5C reductase (16). The product of all three isozymes is proline in mitochondria and/or cytosol and parametabolically, the cycling of redox and the maintenance of total pyridine nucleotide pools (48) and redox ratios (22). Based on these considerations, a revised proline cycle and its metabolic/regulatory functions can be proposed (Fig. 3). However, the recognition that PYCR1/2 are associated with mitochondria requires revision of some aspects of cycling and redox shuttling. The production of P5C from proline as well as from glutamate and ornithine is all located in mitochondria. The conversion of P5C to proline by PYCR1/2, however, is associated with activation of glycolysis in the cytosol (48). We could propose that PYCR1 is associated loosely with the mitochondrial membrane catalyzing the cytosolic oxidation of NADH. More likely, there may be channeling or shuttling of NADH and NAD^+^ by established (58) or novel shuttle mechanisms. It is also possible that the location and function of a specific reduced pyridine nucleotide may vary by cell type, its microenvironment, and metabolic (stress) context (15) (see the Proline Synthesis and Cell Regulation section below).

### PRODH/POX-dependent signaling

The localization of PRODH/POX to mitochondria and the donation of proline-derived electrons to complex II of the electron transport chain led to the demonstration that succinate dehydrogenase and PRODH/POX are coregulated (30). However, beyond direct interaction between PRODH/POX and the TCA cycle, the regulatory role of proline degradation was suggested by the discovery that PRODH/POX, designated p53-induced gene 6 (PIG-6), was rapidly and robustly induced by adeno-P53 (82). In subsequent studies (Fig. 2), PRODH/POX was induced not only by p53 and PPARγ (68, 78) but also by AMPK (47). The mechanism for downstream signaling was mediated by the generation of mitochondrial ROS (53). The downstream effects of ectopic overexpression of PRODH/POX in colorectal cancer cells stably transfected with a Tet-off PRODH expression vector include downregulation of DNA synthesis, blockade of the cell cycle, and decrease in the phosphorylation-dependent steps of the MAP kinase pathway and apoptosis (78).

Although the initial observation was in the context of apoptosis and KD of PRODH/POX inhibited apoptosis induced by cytotoxic stimuli, subsequent studies showed that PRODH/POX was involved in other cellular processes. Stimulated by oxidized low-density lipoprotein, nutrient deprivation, or hypoxia, PRODH/POX was involved in the initiation of autophagy (50). Whether the endpoint was apoptosis or autophagy depended on the cellular and metabolic context, but an essential component of the signaling pathway included the activation of PRODH/POX. Finally, Pandhare *et al.* showed that in neuronal cells, the glycoprotein 120 (gp-120) of human immunodeficiency virus 1 (HIV-1), binding to the surface receptor CXCR4, activated P53, which induced the expression of PRODH/POX (69). The ROS thus generated led to autophagy.

Recent studies showed the critical role of PRODH/POX in cellular senescence (64, 65). Senescence is defined as the state of permanent cell growth arrest, and although this process is related to apoptosis and is induced by p53, it has mechanisms that are distinct. The maintenance of cells in a metabolically active but permanently nonproliferative state may be of importance especially in cancer. Using fibroblasts titrated with low concentrations of etoposide to damage DNA and senescence-activated β-galactosidase as a marker, workers identified senescence genes before the expression of apoptotic genes. They found that *PRODH* is one of four genes identified by differential transcriptomics; it is one of two genes also induced by p53 and one of three genes when ectopically expressed could bring on senescence. It is likely that PRODH/POX plays a critical role in stress-related senescence. In a subsequent publication, these workers showed that it is ROS generated by PRODH/POX which is the critical initiator of senescence (65).

The role of PRODH/POX and the proline cycle may be reprogrammed to serve various functions depending on the cellular and microenvironmental context. In a model for metastasis of breast tumors, Elia *et al.* reported that in a 3D spheroidal cell culture model, PRODH/POX was threefold higher than the same cells in two-dimensional growth (22). Under these conditions, PRODH/POX was important in the maintenance of ATP by a mechanism independent of NADH. Proline cycling, that is, the production of proline by PYCR1 to serve as substrate for PRODH/POX was implicated.

### PRODH/POX in animal models

**FIG. 4.**
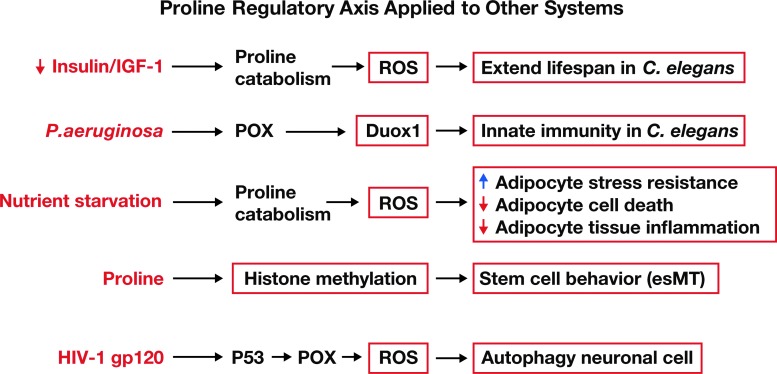
**Recently described regulation based on proline metabolic axis.** References: “Extended lifespan” (106); “Innate immunity” (91); “Adipocyte stress resistance” (44); “Stem cell behavior” (11); “Autophagy neuronal cell” (69). Duox, dual oxidase; esMT, embryonic stem cell-to-mesenchyme-like transition (for details see text); HIV, human immunodeficiency virus; IGF, insulin-like growth factor.

Although this review emphasizes the role of the proline metabolic axis in human cancer, findings in several other experimental models are of interest and support the validity of the central regulatory role of the proline metabolic axis (Fig. 4). In *Caenorhabditis elegans*, Zarse *et al.* showed that PRODH is the linking signal between dysfunctional insulin signaling and longevity (88, 106). She used *C. elegans* with a defect in its insulin receptor analog and mouse embryonic fibroblasts with dysfunctional glucose transport. In both cases, glucose utilization was compromised and interestingly, PRODH/POX was upregulated. The ROS produced from stress-induced proline oxidation caused cells to increase the production of antioxidant enzymes that prolonged survival. Recently, increased expression of PRODH was found in *C. elegans* challenged with *Pseudomonas aeruginosa* (91). The ROS derived from PRODH/POX activated dual oxidase (Duox 1), an enzyme known to be important in innate immunity (91). Other workers using a mouse model showed that aging animals have diminished blood supply in adipose tissue, and fat cells become nutrient deprived such that they must metabolize stored lipid to survive. PRODH/POX is critical for the induction of adipocyte triglyceride lipase (44). Although these are interesting studies that stimulate conceptual advances, an extensively studied system is that described for developing ESCs (see the Development in ESCs section below).

## P5C Activates Cells in Tissue Culture

Although PRODH/POX plays a regulatory role by generating ROS for redox signaling, P5C is also a product that can be recycled to proline by PYCRs with its regulatory properties. In the context of recent discoveries, previous work describing unusual metabolic features of P5C may provide mechanistic insights. At low micromolar concentrations, P5C added to cultured cells robustly increased the activity of the oxPPP as measured by the generation of ^14^CO_2_ from glucose-1-^14^C (74, 76, 101). This was seen at all concentrations of glucose studied, and at saturating concentrations of P5C, the activity of the oxPPP was increased nearly 15-fold (102). We considered that this effect was due to the metabolism of P5C by PYCR in a metabolic interlock with glucose 6-phosphate dehydrogenase (G6PD) and 6-phosphogluconate dehydrogenase (6PGD). In human red cells free of mitochondria but with robust P5C reductase activity, not only is the oxPPP activated by P5C but the production of ribose-5-phosphate (R-5-P), phosphoribosyl pyrophosphate (PRPP) and the activity of the salvage pathway for purine ribonucleotides were all robustly increased (103, 104). Importantly, the linkage with P5C was lost in cells deficient in G6PD (104). Other workers translated these P5C effects on R-5-P and PRPP to mouse liver *in vivo* (6).

That P5C through the catalytic activity of PYCR could be a useful activator of nucleotide salvage was an interesting possibility, but the physiologic importance was not understood until recently (see below). P5C has been shown to be an extracellular metabolite and although concentrations in plasma are in the low micromolar range (25), it can be found in millimolar concentrations in certain inborn errors (Type 2 hyperprolinemia) (26). Furthermore, plasma P5C undergoes meal-dependent fluctuations (24). Experimental models show that it can be made in one tissue and released into the extracellular fluid and taken up and metabolized by another cell type (81). This can be meaningful in the microenvironmental metabolism of tumors with the cometabolism of tumor cells, stromal cells, and red cells. Thus, P5C from a variety of sources, proline, glutamate, ornithine, or taken up (63) from the circulation (25), may play a role in regulation.

### Oxidative pentose phosphate pathway

**FIG. 5.**
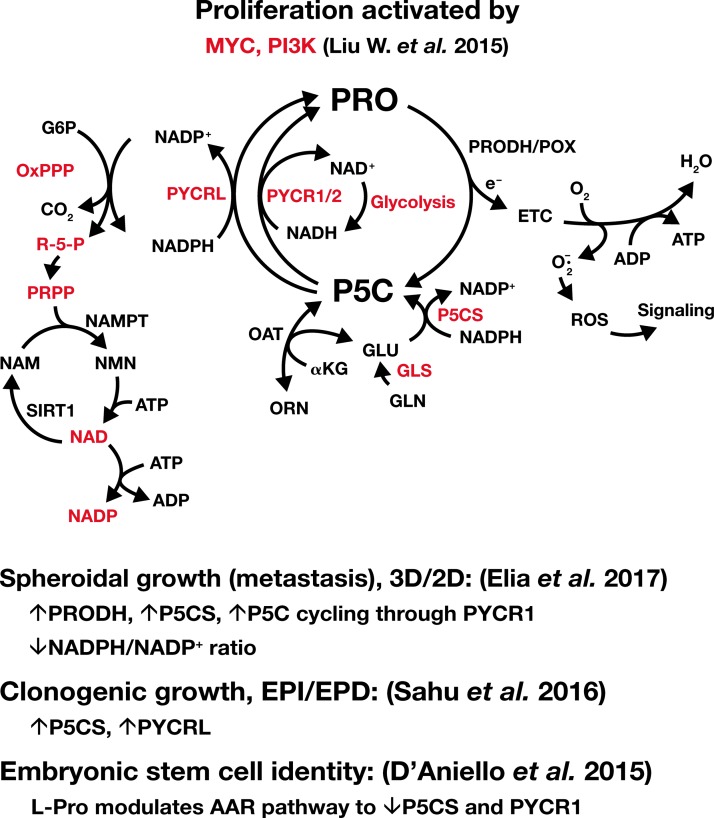
**Pathways activated by MYC and PI3K.** Enzymes or pathways upregulated by MYC and PI3K are shown in *red*. Abbreviations are as shown in figures 1–3. NAD, total nicotinamide adenine dinucleotide; NADP, total nicotinamide adenine dinucleotide phosphate; NAM, nicotinamide; NAMPT, nicotinamide phosphoryl transferase; NMN, nicotinamide mononucleotide; OxPPP, oxidative arm of pentose phosphate pathway; PRPP, phosphoribosyl pyrophosphate; R-5-P, ribose-5-phosphate; SIRT1, sirtuin 1.

More fully discussed in the Proline Synthesis and Cell Regulation section, the metabolic interlock between PYCR and the oxPPP is an important junction for metabolic regulation. As shown by Eggleston and Krebs, the flux through the oxPPP depends not only on the levels of G6PD and 6PGD but also by the levels of NADP^+^ and NADPH (21), a point emphasized in a recent review (34). Not only is concentration of NADP^+^ important as the cofactor for G6PD but the enzyme activity also is inhibited by the reduced form of NADPH competing with NADP^+^ for binding. This may explain the finding in PC9 cells (48), where the KD of P5CS markedly decreased the activity of the oxPPP, but this decrease could be mitigated by the addition of P5C. However, with KD of PYCR1/2/L, the oxPPP was similarly decreased and no compensatory increase was seen with added P5C (48) (Fig. 5). In addition, in tet-off-myelomatosis oncogene cellular homologue (MYC) P493 cells in which MYC expression can be controlled by tetracycline, the addition of P5C with MYC-on could increase the oxPPP activity, whereas with MYC-off, the oxPPP was reduced by about 60% and no longer responded to P5C (48). As mentioned above, the oxPPP is in a metabolic interlock with PYCR and the addition of P5C markedly increased oxPPP, the level of PRPP, and the production of purine nucleotides through the salvage pathway (104). Others have shown that the maintenance of pyridine nucleotides occurs by the pyridine salvage pathway, which takes nicotinamide (NAM) back to nicotinamide mono nucleotide with PRPP as the cosubstrate (107). Our data suggest that the effect of the proline metabolic axis on the glycolytic pathway as well as the oxPPP may be due to the maintenance of total pyridine nucleotides NAD and NADP. This hypothesis based on the proposed metabolic interlock will require additional studies (Fig. 5).

**FIG. 6.**
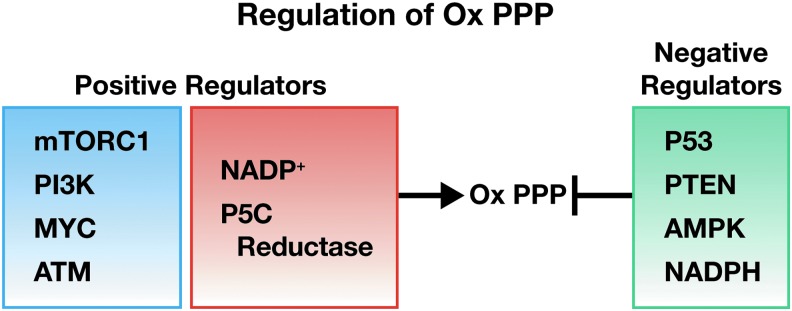
**Regulation of the oxidative arm of the pentose phosphate pathway, adapted from Jiang** ***et al.*** **(34).** AMPK, AMP-activated protein kinase; ATM, ataxia telangiectasia mutated protein kinase; mTORC1, mammalian target of rapamycin1; MYC, myelocytomatosis oncogene cellular homologue; PI3K, phosphoinositide 3 kinase; PTEN, phosphatase and tensin homologue.

The oxPPP has been emphasized as a pathway for producing reducing potential in the form of NADPH (34). Certainly, NADPH is critical for reducing glutathione, for other redox transfers, and for lipid synthesis. Thus, oxPPP has an important role for resisting oxidative stress (34). On the contrary, the production of ribose and PRPP for nucleotides has been assigned to the nonoxidative PPP (non-oxPPP) (43). The conclusion has been based on higher levels of transketolase and transaldolase in cancer cells, which can divert and rearrange intermediates from the glycolytic pathway to form ribose-5-P, the precursor of PRPP (7, 70). Although the oxPPP is robustly upregulated by oncogenes (Fig. 6), the flux to PRPP seems to favor the non-oxPPP (43).

Two possible considerations may have led to this conclusion. First, many established tumor cell lines are grown in media with supraphysiologic concentrations of glucose, for example, Dulbecco's modified Eagle's medium contains glucose at 4.5 g/L or 450 mg/dL (25 mM), much higher than physiologic concentrations of 100–150 mg/dL (5–8 mM). Thus, cultured cells have been selected or “addicted” to grow with a glut of glucose, and intermediates in glycolysis (Warburg effect) can be diverted to the non-oxPPP. Importantly, under stress conditions or with cells in transition, for example, from quiescence to the proliferative mode, the oxPPP is activated with upregulation of G6PD and 6PGD. With the concomitantly upregulated PYCRs as a source of NADP^+^ (Fig. 6), the oxPPP could rapidly and robustly yield R5P and PRPP (6, 74, 101) (see the P5C Activates Cells in Tissue Culture section above). The latter is a critical intermediate in these transitions for producing pyridine nucleotides as well as purine and pyrimidine nucleotides. With KD of P5C synthase or all three PYCRs, the level of total NAD and total NADP markedly decreased, as did glycolysis and proliferation (48).

## Proline Synthesis and Cell Regulation

The last 18 months have seen a flurry of publications on the role of proline biosynthesis in cell regulation (22, 35, 48, 55, 86). The discovery that the enzymes of the proline biosynthetic pathway (P5CS, PYCR1/2/L) were markedly upregulated by c-MYC (49) provided an impetus. The coupling to regulatory networks suggested a function more novel than just the proline requirement for protein synthesis. Such a possibility was pursued by several laboratories using a variety of approaches.

Focusing systematically on metabolic endpoints, Liu *et al.* first showed that proliferation rates were markedly affected by KD of proline biosynthetic enzymes. In Tet-off-MYC P493 and in PC9, a lung cancer cell line expressing high levels of endogenous MYC and proline synthetic enzymes (49), KD of MYC or proline enzymes markedly decreased growth. The addition of proline or P5C partly corrected the decrease in proliferation due to KD of P5CS, as well as with KD of PYCR1/2, but could not mitigate the effect with the KD of PYCRL. With P5CS KD, the production of P5C and proline from glutamate was blocked, but interestingly, PRO and P5C both could alleviate the effect produced. However, if the KD of P5CS was accompanied by the simultaneous KD of PRODH/POX, the effect of PRO was markedly attenuated, but the recovery effect with addition of P5C was unaffected by KD of PRODH/POX. These studies suggested that an important function of PRODH/POX was to produce P5C to be recycled by P5C reductase (Figs. 3 and 5).

Liu *et al.* also showed that rates of glycolysis in P493 cells were markedly increased by expression of c-MYC (48). The addition of exogenous P5C further increased the rates of glycolysis. In addition, KD of P5CS or the PYCRs markedly decreased the MYC-stimulated rates of glycolysis. The oxPPP was also markedly decreased with KD of P5CS and PYCRs. These effects are consistent with the proposal that the conversion of P5C to proline is coupled to both NADH and NADPH redox cycling, but no consistent shift in redox ratios was seen. Instead, there was a striking decrease in total NAD and total NADP when P5CS was knocked down. KD of individual PYCRs had little effect, but KD of all three PYCRs resulted in marked decrease in both NAD and NADP and the effect was not mitigated by the addition of either proline or P5C (Fig. 5). Presumably, the function of the PYCRs is necessary, but there is functional redundancy among the three isoforms. The maintenance of pyridine nucleotide pools has been emphasized by others (107). The question then arises—how do the activities of PYCRs affect the levels of total NAD and total NADP?

We proposed that the proline metabolic pathway and the proline cycle function to not only produce proline for protein synthesis but to also mediate redox regulation through parametabolic mechanisms (77, 79). Revealing publications were those by Loayza-Puch *et al.* (55, 56), which addressed a related question by monitoring amino acid deficiencies using differential ribosome codon reading (*diricore*) based on the accumulation of ribosomes at a particular codon, suggesting deficiency of the corresponding amino acid. Comparing clear cell carcinoma tissues *versus* normal kidney tissues from the same patient, the *diricore* showed that the tumor was deficient in proline for protein synthesis. It is unlikely that the proteinogenic demand for proline in cancer is greater than for other amino acids. Instead, one can speculate that sequestered proline regulatory functions, for example, in mitochondria, may have priority in cancer over proline for protein synthesis. Elia *et al.* (22) recently reported that in MCF10A, H-RasV12 grown in 3D spheroids activates proline degradation and proline cycling. Intracellular levels of proline were markedly decreased, a finding consistent with the hypothesis that proline is diverted away from protein synthesis for critical regulatory functions.

Another recent publication addressed the role of proline biosynthesis in melanoma cells. Kardos *et al.* identified ALDH18A1, the gene encoding P5CS in an inhibitory RNA (RNAi) screen of a kinase library, as critically important for cell growth (35). Knocking down P5CS decreased melanoma cell viability and tumor growth. In the face of P5CS KD, the addition of 1.25–2.5 mM proline to the medium only partially mitigated the decrease in cell viability, suggesting that P5CS is mediating effects other than producing product proline. Of the two known regulatory systems for amino acids, that is, the mechanistic target of rapamycin (mTOR) axis and the general control non-depressible 2 (GCN2) system, these authors showed that mTOR is not involved in the effects seen with P5CS KD. Although GCN2 is implicated by this finding that phosphorylation of erthythrocyte initiation factor 2 α (eIF2α) is increased in some melanoma cells, the process requires prolonged treatment suggesting indirect effects rather than directly due to proline depletion. To quote the authors, “It is also possible that knockdown of P5CS affects more processes than just protein synthesis.” (35) We would agree (Fig. 5). Nevertheless, the identification of ALDH18A1 encoding P5CS as a critical gene for melanoma viability and growth was an important finding.

Two important articles by Sahu *et al.* (86) and Elia *et al.* (22), respectively, showed how reprogramming of the proline metabolic axis occurs under altered nutritional or microenvironmental conditions (Fig. 5). Sahu *et al*., using clonogenicity rather than proliferation as the endpoint (86), screened a subset of cancer cells and formed the dichotomy of EPD and EPI (exogenous proline dependent and independent, respectively) cells using RPMI (with proline) and DMEM (no proline). The enzyme complement differed in these two populations. The EPI cells had higher amounts of P5CS and P5CR3 (PYCRL) and higher levels of c-Myc. It is interesting that PYCRL is the isozyme permissive for EPI since it preferentially uses NADPH and is coupled to the oxPPP (see the Oxidative pentose phosphate pathway section above). With KD of PYCRL, the addition of proline only partially restored the clonogenicity to normal (∼50%). They also tested the well-known mTOR system and found that the phosphorylation of the downstream product 4EBP1 was elevated in EPI compared to EPD. Inhibition of 4EBP1 as well as p70S6K1/2 downstream products of mTOR inhibited clonogenicity. The authors propose that under clonogenic conditions, proline metabolism is required to relieve ER stress, and they speculate that proline metabolism is required to help manage stress during tumorigenic growth.

Using 3D spheroid culture as a model for metastasis of breast cancer, Elia *et al.* showed that proline metabolism is reprogrammed (Fig. 5). As mentioned in the PRODH/POX-Dependent Signaling section, spheroids in 3D have markedly higher PRODH/POX than the same cells grown in monolayer (2D). Furthermore, they showed that cycling of proline helped maintain ATP levels and growth. However, PYCRs were necessary even in the face of added proline, and the intracellular levels of proline were markedly decreased, a finding consistent with the findings of Loayza-Puch using *diricore* (55, 56). In 3D cultures, with high PRODH/POX and recycling, the NADPH/NADP^+^ ratios were markedly decreased. Thus, they concluded that PRODH/POX played a critical role for generating energy under conditions of 3D culture and the PYCRs played a role in the redox maintenance of pyridine nucleotides. Using l-tetrahydro-2-furoic acid (THFA) to inhibit PRODH/POX, these workers showed an inhibition of metastasis using an orthotopic injection of breast tumor cells. These findings are important not only in introducing a novel strategy to control metastases in breast cancer but also in supporting the concept of proline cycling and its parametabolic effect (77).

In a seminal article translating the findings from the proline metabolic axis to studies of patients with breast cancer, Ding *et al.* analyzed PYCRs and compared PYCR1 with PYCR2 in 139 new patients as well as data from 2353 assessable breast cancer cases (17). They showed that PYCR1 was expressed significantly higher in breast cancers with molecular subtypes with poor outcome. PYCR1 but not PYCR2 correlated with poor survival. This was the first clinical demonstration that proline metabolism is relevant to human cancers. Although the findings in this article focused on breast cancer, future research may find additional tumors that may be dependent on proline metabolism in various reprogrammed configurations.

## Development in ESCs

With the recognition of the importance of cancer stem cells, mechanisms governing the development of ESCs provide useful insights into stress-mediated cellular transitions. Interestingly, the proline metabolic axis plays an important role in these transitions. In ESCs, a role for proline was first described by Washington *et al.* (97), expanded by several reports from Minchiotti's laboratory in Naples (8, 11–13) and recently reviewed by Kilberg *et al.* (36). The original observation was that proline in the culture medium augmented proliferation and differentiation of mESCs (mouse ESCs) into epistem cells (EpiSCs) or primitive ectoderm (PrEc). These proline-induced cells (PICS) were shown to differentiate into general mesendoderm (97). The Naples group then found that ornithine could also induce the mESCs to EpiSCs implicating P5C as well as proline as the common intermediate (11). It is tempting to speculate that P5C is the downstream metabolite mediating these effects especially with recent emphasis of the proline-P5C cycle. An important finding by Comes *et al.* (11) is that proline treatment resulted in the global increase in H3K9 and H3K36 histone methylation at genomic regions with increased methylation during the blastocyst to epiblast transition, suggesting that the proline-induced cells correspond to a fully reversible early primed pluripotent state (13). The Naples group also found that l-Pro may mediate its effects through the Gcn2-Eif2a-Atf4 amino acid starvation response pathway. They described an autoregulatory loop by which the l-Pro induced ESC proliferation and ESC mesenchymal transition (esMT) is inhibited by halufinogen (12), a specific inhibitor of prolyl-t-RNA synthetase. The overexpression of activity transcription factor 4 (Atf4) will induce the proline biosynthetic pathway, that is, P5CS and PYCR1 (12). Although they showed that this pathway appears independent of PRODH-generated ROS, involvement of PRODH-produced-P5C (proline cycle) remains a possibility (see above, Fig. 3).

## Protein–Protein Interactions

**FIG. 7.**
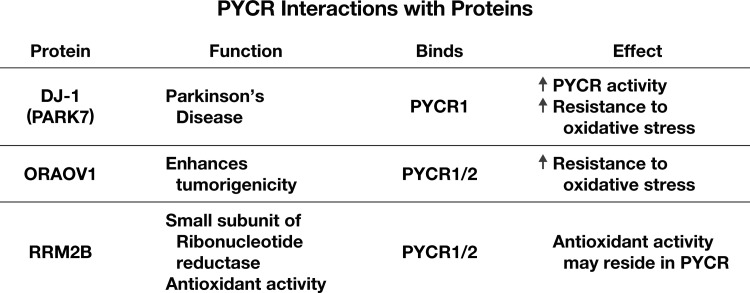
**Protein–protein interactions with PYCR.** References: DJ-1 (100); ORAOV1 (92); RRM2B (39). DJ-1, protein deglycase; ORAOV1, oral cancer overexpressed 1; PARK-7, Parkinson's disease protein 7; RRM2B, ribonucleoside-diphosphate reductase subunit M2B.

A number of observations of the proline regulatory axis suggest compartmentation and/or channeling of substrates (87). Several reports have described binding and functional interaction of a specific protein with PYCRs, which may provide mechanisms for decoding novel regulatory mechanisms (Fig. 7). Examples are the binding of DJ-1 (PARK7), a Parkinson disease protein, to PYCR1 (100). Oral cancer overexpressed (ORAOV1), a protein associated with aggressiveness and invasiveness of tumors, binds to PYCR1/2 (92). The physiologic significance of these interactions requires additional elucidation. Nevertheless, one specific report is noteworthy because it involves RR, the enzyme catalyzing the conversion of ribonucleoside diphosphate to deoxyribonucleoside diphosphate (dNTPs), a critical step for DNA replication and repair. The holoenzyme of RR is composed of two large subunits, RRM1, and two small subunits, RRM2 or RRM2B (40). Kuo *et al.* found that PYCR1 and PYCR2 bind RRM2B thereby linking DNA replication and repair to proline synthesis (39). Of special importance, RRM2B is low under unstressed conditions but is induced by stress such as DNA damage or oxidative stress (40) and is associated with antioxidant activity. With convincing evidence that RRM2B binds to either PYCR1 or PYCR2, they showed that the KD of PYCR1/2 negated the antioxidant effect of RRM2B. These studies suggest that RRM2B acts as a signal to alter the function of PYCR1/2. The question whether RRM2B and PYCR isozymes regulate each other's respective catalytic activity remains unanswered. Since others have shown that PYCR augments redox cycling (22, 48) and helps to maintain pyridine nucleotides presumably by providing substrates for salvage pathways (48, 104), the linking of this activity to the supply of dNTPs for DNA replication and repair supports the hypothesis that proline synthesis is linked to mechanisms for regulating proliferation and/or DNA repair.

## Inborn Errors of the Enzymes of Proline Synthesis

Inborn errors of metabolism in humans provide insights into mechanisms as well as opportunities to validate specific hypotheses. Although there are inborn errors of proline metabolism involving enzymes along each step of the degradative and synthetic steps of the pathway, the proline synthetic enzymes are emphasized in this review.

### P5CS

Baumgartner *et al.* reported the first inborn error in proline biosynthesis as due to a defect in P5CS, the first enzyme in the pathway from glutamate to proline (4). Two affected siblings with a missense mutation (R84Q) showed relatively low levels not only of proline but also of ornithine, citrulline, and arginine. The urea cycle intermediates are low because P5CS catalyzes the only direct pathway between the TCA cycle and urea cycle. The functional defect was shown indirectly by measuring ^3^H-glutamate incorporation as proline in protein, and the clinical phenotype included hypotonia, dysmorphic signs, *pes planus*, and clonic seizures (4). Both siblings developed progressive neurodegeneration, joint laxity, and skin hyperelasticity. Since subsequent studies in patients with PYCR deficiency showed abnormalities in redox defense, it would have been interesting to examine whether these patients or their cells had altered resistance to oxidizing challenge. Nevertheless, the neurodegeneration and seizures may be related to redox dysregulation.

### PYCR1

Reversade *et al.* described PYCR1 deficiency in 35 affected individuals with *cutis laxa* and progeroid features (85). These features included wrinkly skin and bone loss giving patients an aged appearance. There is also osteopenia, mental retardation, and abnormal corpus callosum in some individuals. Mitochondria were found to be abnormal in cultured skin fibroblasts from subjects studied. Interestingly, the fragmentation of mitochondria was increased in affected cells exposed to H_2_O_2_. Thus, the deficiency in PYCR1 caused cells to be less resistant to oxidizing challenge.

### PYCR2

Two affected siblings in two consanguineous families were shown to have mutations in PYCR2 with microcephaly and hypomyelination as the prominent clinical features (66). Unlike the patients with PYCR1 deficiency, the PYCR2-deficient patients did not have *cutis laxa* or osteopenia. Thus, the distinct phenotypes suggest that these two isozymes with some biochemical distinctions may be developmentally responsible for these differences. Interestingly, plasma amino acid analysis in two individuals did not show a decrease in plasma proline. Thus, the authors suggested that “deficiency of proline, as a building block of proteins, might not be the major pathophysiology.” (66) These workers did find, however, a metabolic abnormality in redox regulation. In cultured cells exposed to 400 mM H_2_O_2_ for 1 h, followed by 24 h in culture, control cells showed a modest increase (approximately twofold) in TUNEL-positive cells as an assessment of apoptosis, whereas PYCR2 mutant cells showed a nearly sixfold increase in TUNEL-positive cells. Thus, in the area of resistance to oxidizing insult, both PYCR2 and PYCR1 mutants showed similarities even though their phenotypes were different. These findings in humans with inborn errors strongly support the concept that it is not proline for protein synthesis alone that is affected by PYCR mutations, but critical redox regulatory functions are deficient.

The mechanisms for the loss of resistance to oxidizing insult remain mechanistically uncertain. However, a common denominator may be the failure to maintain adequate levels of total pyridine nucleotides. In *Saccharomyces cerevisiae*, Liang *et al.* found that proline biosynthesis is required for endoplasmic reticulum stress tolerance (45). Although the PYCRs can recycle redox, the deficiency of PYCRs seems inimical to maintaining antioxidant activity. However, a common denominator may be the inability to upregulate the level of total pyridine nucleotides, both NAD and NADP, under challenge by oxidants. The PYCRs are linked to the oxPPP (48), and the generation of PRPP necessary for nicotinamide phosphoribosyltransferase (NAMPT), the salvage pathway for pyridine nucleotides. When challenged, the total content of NAD and total NADP may be inadequate for maximizing the turnover of the glutathione cycle, thereby resulting in the decreased resistance to oxidizing challenge.

## Conclusion

Recent discoveries have solidified the importance of the proline regulatory axis not only in cancer but also in other metabolism-dependent models, that is, aging, senescence, and development of ESCs. These reports have provided support for both the proline cycle and the parametabolic functions of the proline metabolic axis. Of special interest is the adaptation of proline metabolism to the cellular context and microenvironment. Nevertheless, the new findings demand revisions in the existing model, especially since PYCR1/2 may be mitochondrial. Work from KD studies of each or all the enzymes of proline biosynthesis suggests that they augment redox cycling. In addition, by stimulating the oxPPP, the proline cycle activated by MYC and PI3K increases the level of total pyridine nucleotides. This may augment redox cycling not only for the Warburg effect but also to increase the capacity for antioxidant defenses, a feature identified in inborn errors of the proline synthetic enzymes, which at first appeared paradoxical. The identification of inadequate proline for protein synthesis based on *diricore* studies suggests that in cancer tissue these regulatory mechanisms may take precedence over supplying proline for protein synthesis. Thus, we have gained insight into the regulatory effects not only of proline metabolism but also the metabolism of other NEAA, which may lead to new strategies for metabolic therapeutics.

## Abbreviations Used

3D: three-dimensional
4EBP1: 4 erythrocyte binding protein 1
6PGD: 6-phosphoglulconate dehydrogenase
AMPK: AMP-activated protein kinase
Atf4: activity transcription factor 4
ATM: ataxia telangiectasia mutated protein kinase
ATP: adenosine triphosphate
*Diricore*: differential ribosome coding reading
dNTP: deoxyribonucleoside diphosphate
Eif2α: erythrocyte initiation factor 2 α
EpiSCs: epistem cells
ESC: embryonic stem cells
esMT: embryonic stem cell mesenchymal transition
ETC: electron transport chain
G6PD: glucose-6-phosphate dehydrogenase
GCN2: general control non-derepressible 2
gp 120: glycoprotein 120
GSA: glutamic -γ-semialdehyde
HIV-1: human immunodeficiency virus 1
hsERV: human-specific endogenous retrovirus
IGF: insulin-like growth factor
KD: knockdown
mESCs: mouse embryonic stem cells
miR-23a/b: microRNA-23 a/b
miR-23b*: microRNA-23b star
mTOR: mechanistic target of rapamycin
mTORC1: mTOR complex 1
MYC: myelomatosis oncogene cellular homologue
NAM: nicotinamide
NAMPT: nicotinamide phosphoribosyltransferase
NEAA: nonessential amino acid
NMN: nicotinamide mononucleotide
non-oxPPP: nonoxidative PPP
OAT: ornithine aminotransferase
ORAOV1: oral cancer overexpressed 1
OxPPP: oxidative arm of the pentose phosphate pathway
P53: tumor protein 53
P5C: pyrroline-5-carboxylate
P5CDH: pyrroline-5-carboxylate dehydrogenase
P5CS: pyrroline-5-carboxylate synthetase
PARK7: Parkinson 7
PI3K: phosphoinositide 3-kinase
PICS: proline-induced cells
PIG-6: p53-induced gene 6
POX: proline oxidase
PPARγ: peroxisome proliferator-activated receptor gamma
PrEc: primitive ectoderm
PRODH: proline dehydrogenase
PRPP: phosphoribosyl pyrophosphate
PTEN: phosphatase and tensin homologue
PYCR1/2/L: pyrroline-5-carboxylate reductase 1/2/L
R-5-P: ribose-5-phosphate
RNAi: inhibitory RNA
ROS: reactive oxygen species
RR: ribonucleotide reductase
RRM1: ribonucleotide reductase M1 subunit
RRM2: ribonucleotide reductase M2 subunit
RRM2B: ribonucleotide reductase M2B subunit
SIRT1: sirtuin 1
TCA: tricarboxylic acid
THFA: l-tetrahydro-2-furoic acid
